# Cell Progeny in the Olfactory Bulb after Targeting Specific Progenitors with Different UbC-StarTrack Approaches

**DOI:** 10.3390/genes11030305

**Published:** 2020-03-13

**Authors:** Rebeca Sánchez-González, María Figueres-Oñate, Ana Cristina Ojalvo-Sanz, Laura López-Mascaraque

**Affiliations:** Department of Molecular, Cellular and Development Neurobiology, Instituto Cajal-CSIC, 28002 Madrid, Spain; rebeca@cajal.csic.es (R.S.-G.); maria.figueres@gen.mpg.de (M.F.-O.); anacris23@cajal.csic.es (A.C.O.-S.)

**Keywords:** Olfactory interneuron, heterogeneity, StarTrack, development, neural progenitor cell, cell fate, in utero electroporation

## Abstract

The large phenotypic variation in the olfactory bulb may be related to heterogeneity in the progenitor cells. Accordingly, the progeny of subventricular zone (SVZ) progenitor cells that are destined for the olfactory bulb is of particular interest, specifically as there are many facets of these progenitors and their molecular profiles remain unknown. Using modified StarTrack genetic tracing strategies, specific SVZ progenitor cells were targeted in E12 mice embryos, and the cell fate of these neural progenitors was determined in the adult olfactory bulb. This study defined the distribution and the phenotypic diversity of olfactory bulb interneurons from specific SVZ-progenitor cells, focusing on their spatial pallial origin, heterogeneity, and genetic profile.

## 1. Introduction

**Figure 1:**
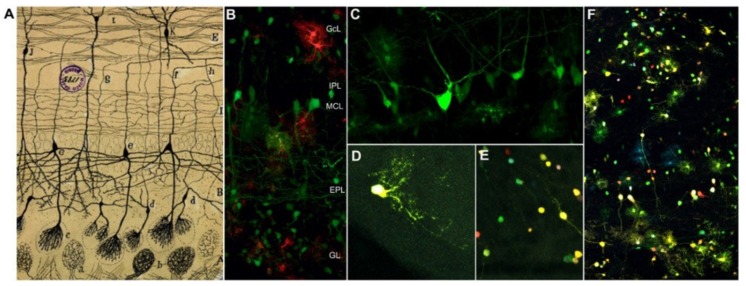
(**A**) Original drawing by Cajal of an olfactory bulb (OB) section from the brain of a perinatal cat [3] showing the glomerular layer (*A*); external plexiform layer (*B*); mitral cell layer (MCL; *C*); internal plexiform layer (*D*); granule cell layer and white matter (*E*); (*a,b*) terminal axonal arborizations of olfactory sensory neurons; (c) dendritic arborizations from tufted (*d*) and mitral cells (e) that form the glomerulus; (*f–h*) axonal projections from tufted and mitral cells); (*I–J*) granule cells; (*K*) short axon cells of the granule cell layer (Cajal Legacy, Instituto Cajal-CSIC, Madrid, Spain). (**B–F**) Adult OB neural cells labeled after in utero electroporation (IUE) of UbC-StarTrack constructs into the E12 mouse embryo lateral ventricle (LV). (**B**) Coronal section of the mouse OB in which UbC-StarTrack labelling shows the different cells that compose the layers described by Cajal. (**C**) Detail of the MCL, with projection neurons and glial cells labeled with UbC-EGFP-StarTrack. Detail of labeled periglomerular (**D**) and granule cells (**E**). (**F**) UbC-StarTrack labeled glia widely spread across the different OB layers. GcL, granular cell layer; IPL, internal plexiform layer; MCL, mitral cell layer; EPL, external plexiform layer; GL, glomerular layer.

The mammalian olfactory system is composed of the olfactory epithelium (OE), olfactory bulb (OB), and olfactory cortex (OC). The rodent OB is organized into six layers that contain distinct cell populations and that are essentially made up of two types of neurons, interneurons, and projection/output neurons [1,2]. Using the Golgi method, Santiago Ramón y Cajal described the layers in the OB and its components more than a century ago (Figure 1A reproduces an original drawing of Cajal [3]; reviewed in [4]). His morphological studies on the OB provided the basis to define the neurons present in this structure and when UbC-StarTrack strategy is compared, the cells labeled in the adult OB following in utero electroporation (IUE) are similar to those drawn by Cajal (Figure 1A–F). Mitral cells (Figure 1(Ae),C) are the first cell type to be born in the rodent OB, between E10 and E13, and with a neurogenic peak around E11 [5]. The axonal projections of mitral cells form the lateral olfactory tract (LOT) and they establish direct contacts with the OC [6,7].

Olfactory interneurons (periglomerular and granule cells) are a diverse group of cells located within the glomerular layer (GL) and granular cell layer (GcL; Figure 1D–E). These interneurons arise from progenitors located within the ganglionic eminences that migrate tangentially to their destination in the OB [8,9]. Neural stem cells (NSCs) in the subventricular zone (SVZ) also give rise to olfactory interneurons during postnatal life, and these progenitors are determined between E13 and E15 [9]. The different kinds of interneurons are generated from embryonic to postnatal stages [10,11,12,13], and their temporal origin defines the interneuronal diversity [14]. Glial cells are also widespread in the different layers of the OB, those found in each layer arising from different or the same progenitors (Figure 1F). For example, some astrocytes surrounding a single glomerulus have been shown to be clonally-related [15]. In the embryo, glial progenitor cells are located in the most rostral part of the lateral ventricle (LV), which corresponds to the olfactory ventricle (OV) [15,16,17]. This complex organization and connectivity of the cells that populate the OB are largely determined during embryonic development [5,18,19], albeit with an additional contribution postnatally [20,21].

To date, different approaches have been used to assess the diversity of OB progenitor pools during development, including the use of fluorescent and lipophilic tracers, viral vectors, immunostaining, and the generation of specific mouse lines. Nevertheless, the heterogeneity of progenitor cells has yet to be fully defined, and more recent single-cell transcriptomic analyses have shed new light on the diversity and potential of progenitor cells [22,23]. Moreover, single-cell lineage tracing revealed the fate potential and lineage progression of some progenitors [24,25,26].

Here, in order to decipher the heterogeneity of progenitor cells, using UbC-StarTrack lineage tracing approaches under the specific regulation of different promoters, we targeted specific progenitors by IUE to analyze the fate potential of NSCs in the adult brain. The determination of specific cell types in the OB can be influenced by either the molecular profile by their progenitors, the age of the embryo, and/or the location of the labeled progenitors. The data we obtained here confirm that some degree of diversity is present in the pool of OB progenitor cells, highlighting the need of performing further single-cell analyses to define the progenitor cell identities required to generate the complex OB cytoarchitecture. We demonstrate that the origin, fate, and targeting of progenitors must be taken into consideration when studying OB heterogeneity.

## 2. Materials and Methods

### 2.1. Mouse Line

C57BL/6 mice were housed at the animal facility of the Cajal Institute. All procedures were carried out in accordance with the guidelines of the European Union on the use and welfare of experimental animals (2010/63/EU) and those of the Spanish Ministry of Agriculture (RD 1201/2005 and L 32/2007). All the experiments were approved by the CSIC Bioethical Committee (PROEX 223/16). The day of visualization of the vaginal plug was considered as embryonic day (E0) and the day of birth as postnatal day (P0). In addition, mice were considered adults from P30 onwards. In all the experiments, a minimum of *n* = 3 animals was considered.

### 2.2. Vectors

StarTrack constructs were designed as described previously [16,17], and different combinations of StarTrack constructs were used separately to target the different profiles of the progenitor cells. The hyperactive transposase of the PiggyBac system (CMV-hyPBase) was used to generate different vectors in which the expression of the transposases was driven by promoters for NG2, GFAP, and GSX2. The cloning of the different hyPBase constructs was performed by Canvax Biotech, and the source of the promoters is indicated in Table 1. All plasmids were sequenced (Sigma–Aldrich; Saint Louis, MO, USA) to confirm successful cloning. This strategy allowed specific progenitors with active gene expression of these promoters at the time of electroporation to be labeled in order to track their full progeny. Plasmid mixtures contained the twelve UbC-StarTrack floxed constructs, a transposase of the PiggyBac system under the control of the selected specific promoter (either CMV, NG2, GFAP or Gsx2), and the CAG-CreERT2 vector to remove the episomal copies of constructs [17].

### 2.3. In Utero Electroporation (IUE) and Tamoxifen Administration

In utero electroporation was performed as described previously [17,27]. Briefly, the selected plasmid mixture was injected into the LV of E12 embryos with a micropipette and using an ultrasound device (VeVo-770; VisualSonics, Toronto, Canada) and then co-electroporated (three animals per experimental group). The embryos were returned to the dam’s abdominal cavity, which were then monitored for three days. After birth, all the pups were injected with tamoxifen (Tx, 20 mg/ml dissolved in pre-warmed corn oil: Sigma–Aldrich) to eliminate episomal copies of the plasmids and to achieve heritable and stable labelling of the cell progeny [17]. A single dose of Tx (5 mg/40 gr body weight) was administered intraperitoneally (i.p.) to the litter at P5. The mice were analyzed from P30 onwards.

### 2.4. Tissue Processing

All mice were analyzed at adult stages, anesthetizing them with an i.p. injection of pentobarbital (Dolethal 40–50 mg/Kg; Vetoquinol, Alcobendas, Madrid) and then perfused with 4% paraformaldehyde (PFA) in 0.1M phosphate buffer (PB). Subsequently, the brain of mice was removed and postfixed for two hours in fresh 4% PFA and then in PB. Coronal vibratome sections of the brains (50 µm) were obtained and mounted onto a glass slide with Mowiol for storage at 4 ºC.

### 2.5. Imaging

The sections were examined under an epifluorescence microscope (Eclipse E600; Nikon Instruments, Melville, NY, USA), equipped with GFP (FF01-473/10), mCherry (FF01-590/20) and Cy5 (FF01-540/15) filters. Images were then acquired on a TCS-SP5 confocal microscope (Leica Microsystems, Wetzlar, Germany) using a 20x objective (Leica), with the wavelength conformation as described previously (Table 2) [17,21]. Confocal laser lines were maximal around 40% in all samples. Maximum projection images were analyzed using LASAF Leica and Fiji software ImageJ (https://imagej.net/Fiji/Downloads). All stitching and contrast adjustments were performed with LasX software (LasX Industries; St Paul, MN, USA ) and Photoshop CS5 software (Adobe Inc.; San Jose, CA, USA).

### 2.6. Data Analysis

For each experiment, the number of labeled OB cells per section was quantified along the rostrocaudal axis within the OB (32–40 sections per animal). Cells were counted using the manual cell counter plug-in of ImageJ software and the percentage of those cells, located in specific areas, was calculated. The study of 26,685 labeled interneurons in the OB was considered in this approach. For statistics, GraphPad Prism 6.0 (GraphPad, San Diego, CA, USA) was used, and the statistical significance between two groups was assessed with two-tailed unpaired Student’s *t*-tests. For multiple comparison study, one-way analysis of variance (ANOVA) was used. A confidence interval of 95% (*p* < 0.05) was used to determine statistically significant values. Critical values of * *p* < 0.05, ** *p* < 0.01, and *** *p* < 0.001 were adopted to determine statistical differences. Graphs were obtained using GraphPad Prism and CorelDRAW Graphic Suite 2018 (Corel Corporation, Ottawa, Canada).

## 3. Results

### 3.1. The Fate of OB Cells After Targeting Cell Progenitors at Distinct Ventricular Sites

**Figure 2:**
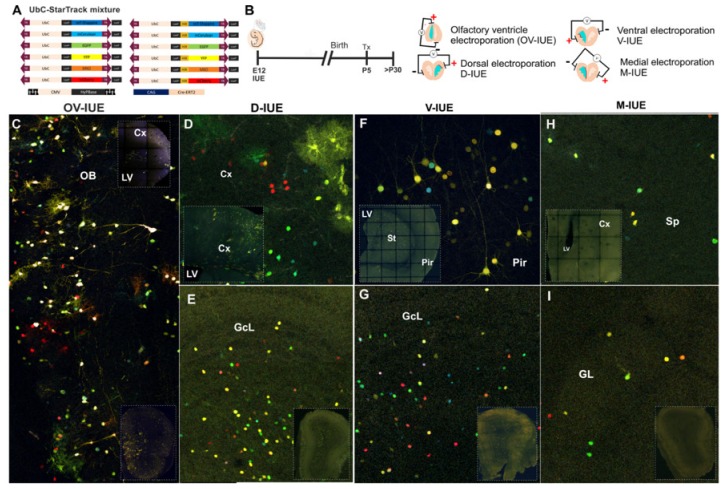
(**A**) Diagram of the UbC-StarTrack vectors, 12 different plasmids encoding six different fluorescent proteins at two different locations, cytoplasmic and nuclear according to the H2B sequence. All vectors were driven by the Ubiquitin C promoter. (**B**) Summary of the IUE procedure, where E12 embryos were injected with UbC-StarTrack mixture and electroporated. After birth Tamoxifen (Tx) was injected at around P5, and the adult tissue was analyzed (>P30). Four different orientations of the electrodes were used for electroporation: olfactory ventricle (OV-IUE), dorsal (D-IUE), ventral (V-IUE), and medial (M-IUE). The red line illustrates the electroporation area. UbC-StarTrack OV-IUE labeled both neurons and glia in the olfactory bulb (OB, **C**). Targeted cells in each lateral ventricular (LV) zone gave rise to different labeled neural cells in the dorsal cortex (**D**), piriform cortex (**F**), and septum (**H**). By contrast, dorsal-, ventral-, and medial- IUE did not produce any labeled glia in the OB; only interneurons were targeted (**E**,**G**,**H**). Dorsal and ventral-IUE targeted progenitors that gave rise to labeled cells in the GcL and eventually, the GL. However, M-IUE produced more labeled cells in the GL. The white squares represent the electroporation area in the telencephalon and OB (**C–I**). IUE, in utero electroporation; OB, olfactory bulb; LV, lateral ventricle; Cx, cerebral cortex; Pir, piriform cortex; St, striatum; Sp, septum.

Using UbC-StarTrack plasmids (Figure 2A), we performed different IUEs at E12 that targeted different ventricular areas (dorsal, ventral, medial) and the most rostral portion of LV, the OV (Figure 2B). Animals were injected with Tx at P5 to remove the episomal copies of the constructs and analyzed at adult stages (from P30 onwards). Rostral IUE, restricted to the rostral OV, labeled glial cells, mitral cells, and some interneurons in the OB (Figure 2C). Interestingly, these glial cells were radially disposed in the different layers of the OB close to the electroporation area. Mitral cells in the mitral cell layer (MCL) were identified through their morphology and the presence of reelin (data not shown). These results indicated that glial and mitral cells originated from progenitor cells located in the most rostral part of the LV at E12. By contrast, when the dorsal, medial, and ventral walls of the LV were targeted, the labeled cells in the OB were periglomerular and granular interneurons, not glial or mitral cells (Figure 2D–I).

After targeting E12 progenitor cells within the dorsal LV, different neural cells were labeled in the adults, spread throughout the corpus callosum and cortex (Figure 2D), although only interneurons were labeled in the OB (Figure 2E). Likewise, ventral electroporation at E12 labeled neurons in the striatum, piriform cortex, and corpus callosum and interneurons in the dorsal cortex (Figure 2F) and the OB (Figure 2G). Dorsal and ventral electroporation mostly labeled interneurons in the GcL, with a few periglomerular cells also labeled. By contrast, medial electroporation labeled cells in the septal area of the telencephalon (Figure 2H), although most cells were located in the GL of the OB (Figure 2I). Finally, IUE of the third ventricle did not label glia or neurons in the OB (data not shown).

In summary, after targeting different ventricular areas at E12, the adult labeled cell-progeny displayed different morphologies at different locations in both OB and forebrain. Thus, the origin of the progenitor cells in specific areas determines their cell fate in the adult telencephalon. 

### 3.2. The Fate of Olfactory Bulb Cells After Targeting Specific Progenitors with StarTrack

**Figure 3:**
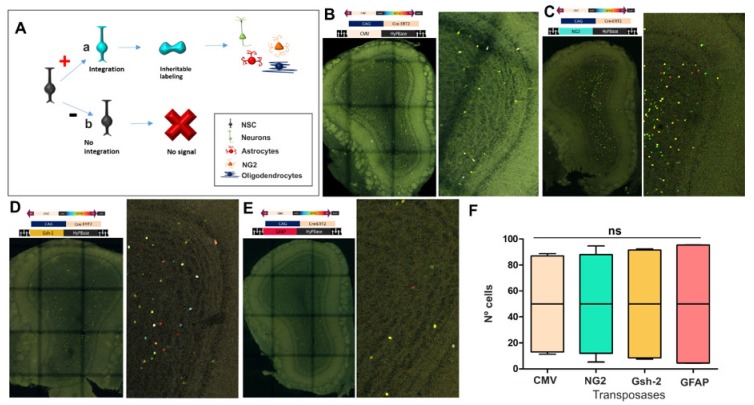
Diagram of the UbC-StarTrack strategy based on transposase promoter expression (**A**). The concept that is focused in the transposase only integrates copies of the UbC-StarTrack vectors into progenitor cells with the corresponding promoter active, labeling all their progeny (**a**). Progenitor cells with the inactive promoter do not integrate copies into the NSCs (**b**). For these experiments, the CMV, NG2, Gsh-2, and GFAP promoters were chosen to target specific NSCs. The first strategy with CMV-hyPBase labeled OB interneurons in the different layers (**B**). The NG2 progeny labeled cells in the GcL and GL (**C**), resembling the Gsh-2 progeny (**D**). GFAP progenitors gave rise to granular cells and periglomerular cells (**E**). GFAP progenitors produced fewer labeled cells in the OB than the other vectors (**F**). All data were normalized; the box plot represents the percentage of labeled cells after targeting each set of progenitors with a specific transposase (whiskers represent 5th/95th percentile, horizontal line displays the median of the data; *n* = 3 for each transposase). Data showed no statistically significant difference between groups (ns). CMV-progenitors are shown in soft pink; NG2-progenitors in blue; Gsh-2 in yellow; GFAP-progenitors in red.

We analyzed the fate of progenitor cells using a novel UbC-StarTrack strategy based on the combination of UbC-StarTrack plasmids with different PiggyBac transposases driven by specific promoters. This strategy drives the integration of the plasmids exclusively into the progenitors that express the specific promoters chosen at the time of electroporation (Figure 3A). As such, we specifically targeted NSCs using the CMV, NG2, Gsh-2, and GFAP promoters. First, the UbC-StarTrack and CMV-transposase (CMV-hyPBase: Figure 3B) incorporated copies of the plasmids ubiquitously, labelling all the progenitor cells and their progeny. Subsequently, the PiggyBac transposase encoding the NG2 promoter (NG2-hyPBase: Figure 3C) was used to target only those progenitor cells with an active NG2 promoter, integrating copies of the plasmids and labeling their progeny. In another approach, the PiggyBac transposase was driven by the subpallial promoter Gsh-2 promoter (Gsh2-hyPBase: Figure 3D) to only label the progenitors located in the ganglionic eminences at early developmental stages and consequently, their adult cell progeny. Finally, the GFAP promoter was incorporated into a transposase (GFAP-hyPBase) and co-electroporated with UbC-StarTrack to label GFAP-progenitor cells (Figure 3E). All these IUEs were directed at the dorso-lateral ventricle walls, except for the Gsh2-hyPBase, which was ventrally orientated. As a result of these manipulations, all the labeled cells in the OB corresponded to interneurons situated in the GL and GcL, with no glial cells or projection neurons. This comparative analysis of the different StarTrack vectors involved 12 animals (*n* = 3 for each transposase driven by a different promoter) and the study of 26,685 labeled interneurons in the OB, of which 12,236 were generated by progenitors expressing NG2; 8308 were from CMV progenitors; 5035 were from progenitors electroporated with the Gsh-2 transposase; and only 1,106 cells were from progenitor cells expressing GFAP. However, no significant differences were evident for each construct in terms of the average of labeled interneurons in the OB (Figure 3F).

Therefore, these results indicate that the pool of progenitor cells committed to give rise to OB interneurons was quite heterogeneous. Accordingly, NG2 and CMV progenitors at E12 produced a larger proportion of adult OB cells compared to those produced from progenitors expressing GFAP. 

### 3.3. Diversity of Olfactory Bulb Interneurons in Relation to Progenitor Cell Identity

**Figure 4:**
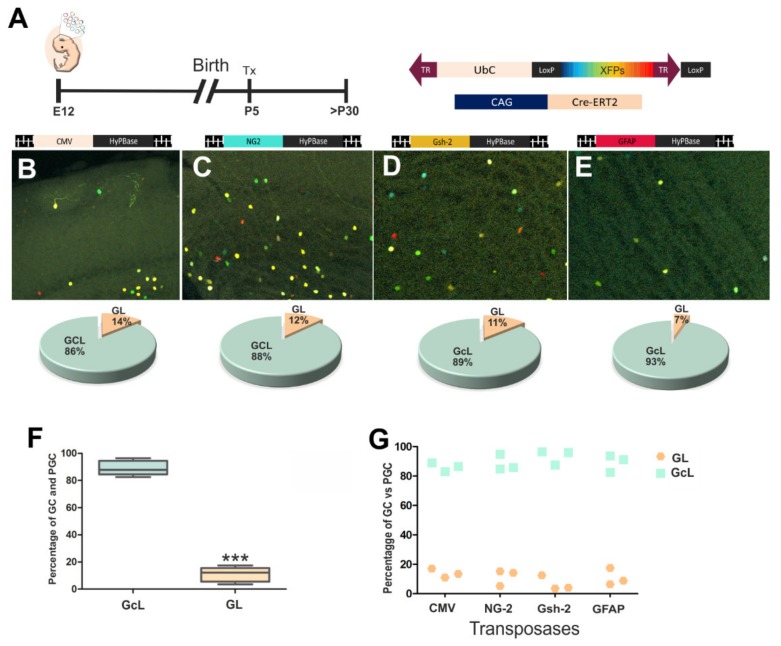
IUE at E12 with the UbC-StarTrack constructs (**A**) and CAG-Cre-recombinase, along with the transposase (**B–E**). All animals were injected with Tx at P5 to remove the episomal copies of the UbC-constructs, and the brain was analysed from P30 onwards. Of the cells produced by CMV-progenitors, 86% were in the GcL and 14% in the GL (**B**). The NG2-progenitors produced 88% GcL cells and 12% GL cells (**C**), similar to the Gsh-2 progenitors (**D**), while GFAP progenitors gave rise to only 7% of GL cells (**E**). Nevertheless, more labeled cells were located in the GcL (green) than in the GL (soft pink). The box plot represents the percentage of labeled cells after targeting each set of progenitors with a specific transposase, and the line displays the mean of the data (box and whisker 5^th^/95^th^ percentile plot). A confidence interval of 95% (*p* < 0.05) was used to determine statistically significant values (***p < 0.001). (**F**). Pallial and subpallial electroporations into the LV produced more cells in the GcL than GL (three animals were analyzed per experiment: CMV; NG2; Gsh-2; GFAP (*n* = 12). Data are shown as average data points (**G**).

Considering the molecular profile of specific NCSs, we studied the differences between the interneurons generated by the different pools of progenitor cells. The UbC-StarTrack plasmids and the CreERT2 vector were injected along with one of the specific transposases (CMV, NG2, GFAP or Gsh-2) (Figure 4A–E). The distribution of the labeled cells in the adult OB was analyzed and correlated with their progenitor cell profile. All the labeled cells were interneurons, periglomerular, and granular cells, even though some immature cells were found close to the subependymal zone (data not shown). Of the cells labeled by the transposase driven by the CMV promoter, 14% were located in the GL, while 86% were located in the GcL (Figure 4B). When NG2 and Gsh2 drove transposase expression, a similar proportion of cells was found in the GcL (88% NG2, 89% Gsh-2) and GL (12% NG2, 11% Gsh-2: Figure 4C,D). However, after targeting the GFAP progenitors, the cell-derived progeny was preferentially sited within the GcL (93%) rather than in the GL (7%: Figure 4E). Besides, these GFAP-progenitors are committed preferably to external areas of GL compared with those that express other promoters. In summary, progenitor cells were committed to preferentially generate granule cells more than periglomerular cells (Figure 4F). Otherwise, there were no significant differences between the distinct types of progenitor cells committed to generate periglomerular and granular cells (Figure 4G). 

Accordingly, these results suggest that E12 progenitors in dorsal and ventral LV produce more granule cells than periglomerular cells, and this cell fate is independent of the molecular profile of NSC.

**Figure 5:**
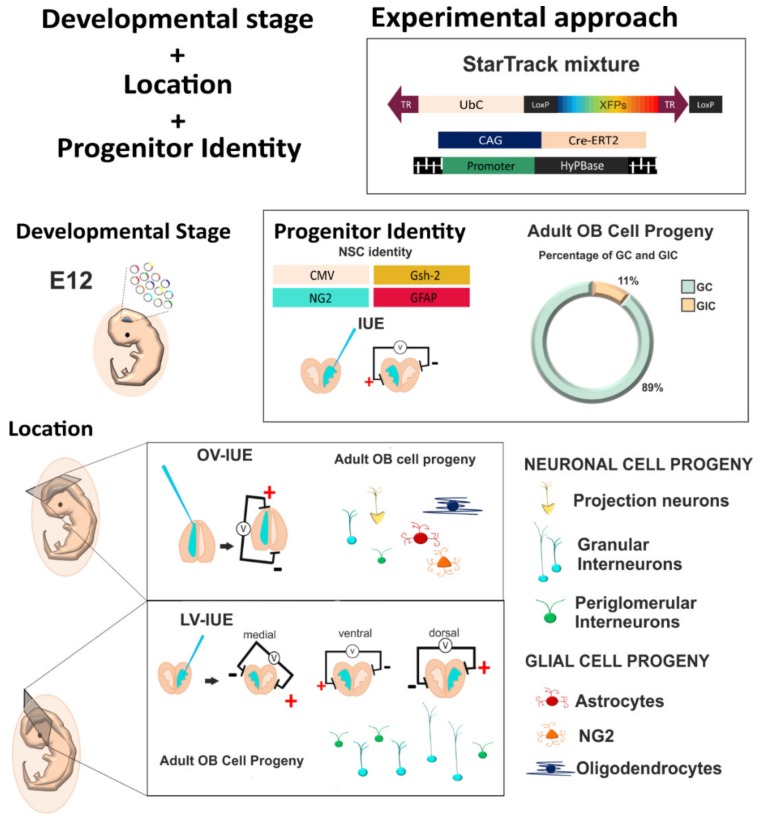
Summary of the importance of age, location, and cell identity to reveal the heterogeneity of NSCs after targeting with the UbC-StarTrack mixture. E12 progenitor cells lining the lateral ventricles can give rise to different neural cell types in the OB depending on their location in the neurogenic/gliogenic niches. The identity of the progenitor is crucial to define the potential fate of the progenitor cells.

In brief, these results summarize the importance of the genes targeted, the location, and the identity of progenitor cells when studying the heterogeneity of specific populations, in this case, adult OB cells (Figure 5). The data obtained open the window for further transcriptomic and clonal studies of these populations in order to define the heterogeneous lineages present in the adult brain.

## 4. Discussion

In this study, a novel StarTrack approach was adopted to address the ontogeny of different cell types in the adult rodent OB, taking into account the identity of their progenitor cells and their location in the LV. We focused on the genetic profile of progenitor cells in order to define the heterogeneity of the NSCs that give rise to neural cells in the adult OB and the origin of these cells. Specific progenitor cells were targeted at E12 to track their adult cell progeny in the OB. StarTrack is a powerful tool to examine the fate and the clonal relationship between cells derived from specific progenitors in vivo, under both physiological and pathological conditions [15,16,17,21,25,27,28,29,30,31,32,33]. This tool also allows progenitor cells to be tracked in vivo, avoiding genetic manipulation of the animals or viral injections. In our particular case, NSCs in the LV were targeted by IUE, avoiding targeting progenitor cells at other sites in which these promoters may be active, such as pericytes in the case of the NG2 promoter [34,35,36]. NG2-hyPBase-driven integration of the StarTrack mix via IUE targets those neural progenitors in the LV with an active NG2-promoter, thereby limiting the developmental spatio-temporal parameters of the study (reviewed by Shimogory and Ogawa [37]).

The heterogeneity of NSC pools is an issue that has yet to be resolved [20,38]. The past two decades have witnessed an accumulation of abundant evidence regarding such heterogeneity, not only in the OB, but also in the dorsal cortex [39], hippocampus [40], and cerebellum [30]. Moreover, recent data show the bipotent capacity of postnatal NSCs to generate OB interneurons and glia in the cortex and striatum [21]. NSCs generating OB cells follow highly patterned and complex behavior during embryogenesis [41,42], and at post-natal stages [13,41]. Single cell analysis provides new insight into the development of the forebrain and the changes it undergoes in the adult. Clonal and transcriptomic analyses make it possible to explore the huge heterogeneity of neural cells [24,32,43,44]. Indeed, the heterogeneity of cortical progenitor cells was recently reported, showing the cell lineage of cortical pyramidal cells restricted to either the deep or superficial layers [39], suggesting that the heterogeneity of neocortical progenitor cells is greater than that previously thought [45,46,47]. Currently, the extent of NSC heterogeneity still remains to be defined as such, and further studies and improved methods will be required to fully specify the cell diversity in the telencephalon.

Thus, it is relevant to target certain sites in order to study specific cell progeny. In particular, the origin of glial cells is not as well understood as that of neurons, and it has been suggested that glial cells travel from the LV to OB via the RMS [48]. The data presented here show that glial and mitral cells progenitors are located in the OV, as previously reported [49]. Nevertheless, it is important to consider those cells in the adult OB that were not targeted by our strategy due to the location of their progenitors, as the ensheathing cells migrating from the olfactory placode to the olfactory nerve layer of the OB [50]. While our data showed the importance of embryonic progenitor location more than the cell identity at E12, neonatal progenitor microdomains may also exist in which specific OB cells originate [13,41].

In summary, the results presented here open a window to explore the genomic profile of neural progenitor cells in more detail. Moreover, they highlight the importance of further studies at the single-cell level to define the heterogeneity of NSC populations and their progeny in the OB. Such studies will help us to better understand this complex brain structure.

## Figures and Tables

**Table 1:** List of the different plasmids used in the StarTrack approach

Vectors	Promoter	Source	Abbreviation
PiggyBac plasmid	Ubiquitin C	Prof. Bradley	UbC-StarTrack
PiggyBac Transposase	CMV	Prof. Bradley	CMV-hyPBase
	NG2	Kirchoff	NG2-hyPBase
	GFAP	Dr Lundberg	GFAP-hyPBase
	Gsx2	Dr K. Campbell	Gsx2-hyPBase
Cre-recombinase	CAG	Dr C. Cepko	Cre-ERT2

**Table 2:** Excitation and emission wavelengths for each fluorescent protein reporter

Wavelength (nm)	YFP	mKO	mCerulean	mCherry	mTSapphire	EGFP
Excitation	514		458	561	405	488
Emission	520–535	560–580	468–480	601–620	520–535	498–514

YFP: Yellow fluorescent protein; mKO: Monomeric kusabira orange; EGFP: Enhanced green fluorescent protein.
